# Powdery Mildew and Aphid Resistance in Wheat–*Thinopyrum intermedium* Derivatives from Zhong Backgrounds

**DOI:** 10.3390/plants15121894

**Published:** 2026-06-18

**Authors:** Qing Guo, Liangxi Zhu, Huihui Wang, Guanlin Liu, Chahong Yan, Yanming Zhang, Yu Sun, Hongjie Li, Lei Cui

**Affiliations:** 1 Shanxi Key Laboratory for Germplasm Innovation and Genetic Improvement in Staple Crop, College of Agriculture, Shanxi Agricultural University, Taiyuan 030031, China; 2 Key Laboratory of Molecular Cytogenetics and Genetic Breeding of Heilongjiang Province, College of Life Science and Technology, Harbin Normal University, Harbin 150025, China; 3 Institute of Biotechnology, Xianghu Laboratory, Hangzhou 311231, China

**Keywords:** adult-plant resistance, alien introgression, multi-stress resistance, pre-breeding germplasm, genotype × environment interaction, phenotypic variation

## Abstract

A total of 159 wheat–*Thinopyrum intermedium* derivatives, originating from six Zhong partial amphiploids, were evaluated for resistance to powdery mildew (*Blumeria graminis* f. sp. *tritici*) at both seedling and adult-plant stages, as well as for field resistance to wheat aphids, together with key agronomic traits. Adult-plant resistance to powdery mildew was common across three years: 34 lines (21.4%) exhibited stable resistance, and 27 (17.0%) were moderately resistant. Resistance frequencies differed among pedigree backgrounds, with Zhong 2 & 5 derivatives showing the highest proportion of stable resistant lines (35.7%). Seedling resistance was detected in 63 lines (39.6%). Aphid resistance was less frequent, with 61 lines (38.4%) classified as resistant, including two highly resistant lines derived from Zhong 3 and Zhong 1 & 3 backgrounds. Combined resistance traits were comparatively rare. Thirty-two lines exhibited resistance to powdery mildew at both seedling and adult-plant stages, while nine lines displayed combined resistance to seedling mildew, adult mildew, and aphids. Analysis of agronomic traits indicated that environmental effects accounted for a substantial proportion of the observed phenotypic variation, whereas pedigree background and resistance responses contributed comparatively little. Correlation analyses revealed generally weak associations between resistance responses and agronomic traits, suggesting that resistance was not a major determinant of agronomic performance within the evaluated population. The identified resistant materials, therefore, represent valuable pre-breeding resources for the incorporation of resistance to multiple biotic stresses in wheat. Further genetic characterization and multi-environment evaluation will facilitate their effective utilization in wheat improvement programs.

## 1. Introduction

Wheat (*Triticum aestivum* L.) is among the most widely cultivated cereals, providing 20% of global calories. Continuous genetic improvement has increased wheat productivity, yet modern wheat improvement has relied heavily on a relatively narrow pool of elite germplasm, resulting in substantial erosion of genetic diversity in cultivated wheat [1,2].

This genetic bottleneck has reduced the availability of novel alleles for resistance to emerging pathogens, insect pests, and environmental stresses, thereby limiting long-term genetic gain and threatening the sustainability of wheat production systems [3,4]. Expanding the genetic diversity of wheat through introgression from wild relatives has therefore become a major objective in modern breeding programs.

Among the major biotic constraints affecting wheat production, powdery mildew and cereal aphids remain destructive and widespread. Powdery mildew, caused by *Blumeria graminis* f. sp. *tritici* (Bgt), is a global foliar disease that causes substantial yield losses by reducing photosynthesis and accelerating leaf senescence [5,6].

Cereal aphids, including *Sitobion miscanthi* and *Schizaphis graminum*, damage wheat through sap extraction, disruption of plant physiological processes, and transmission of viruses such as Barley yellow dwarf virus (BYDV) [7,8]. Aphid infestations can substantially reduce grain yield and quality, especially under favorable environmental conditions and intensive cultivation systems [7]. Climate change and increasing pathogen and pest pressure are further intensifying the economic impact of these biotic stresses in global wheat production [6].

Host resistance remains the most effective, economical, and environmentally sustainable strategy for controlling wheat diseases and insect pests. More than 100 powdery mildew resistance genes and alleles have been identified from wheat and its relatives, whereas comparatively fewer genetic sources of aphid resistance have been characterized and effectively utilized in breeding programs [9,10]. In contrast to fungal disease resistance, aphid resistance in wheat is often quantitatively inherited, genetically complex, and less durable across environments and aphid biotypes [10]. Moreover, resistance to both powdery mildew and aphids is frequently overcome by rapidly evolving pathogen races or insect populations, necessitating continuous discovery and deployment of novel resistance sources [3,4]. Consequently, the identification of germplasm combining resistance to multiple biotic stresses has become an important breeding target.

Wild relatives of wheat constitute an invaluable reservoir of genetic diversity absent from elite wheat germplasm [11,12,13]. Through wide hybridization and chromosome engineering, numerous alien introgressions from species such as *Thinopyrum*, *Secale*, *Aegilops*, and rye have been successfully transferred into wheat, conferring resistance to diseases, insect pests, and abiotic stresses [11,12]. However, the practical utilization of alien introgressions remains challenging. Resistance loci transferred from wild relatives are often linked to undesirable chromosomal segments that negatively affect agronomic performance, a phenomenon commonly referred to as linkage drag [14]. Alien chromatin may also disrupt genome balance and generate instability in yield-related traits, plant architecture, fertility, and adaptation [15,16]. As a result, although many introgression lines possess strong resistance, comparatively few combine resistance with stable agronomic performance suitable for breeding applications [4,12]. The identification of alien introgressions that maintain both resistance and productivity, therefore, remains a major challenge in wheat improvement.

Among wheat wild relatives, intermediate wheatgrass [*Thinopyrum intermedium* (Host) Barkworth & D.R. Dewey; 2n = 6x = 42] is one of the most important sources of resistance to multiple wheat diseases and pests [13]. Through intergeneric hybridization, diverse wheat–*Thinopyrum* derivatives have been developed, including amphiploids, chromosome addition, substitution, and translocation lines [11]. Such introgression materials provide resistance against powdery mildew, stripe rust, leaf rust, stem rust, Wheat streak mosaic virus, BYDV, and aphids [13,17,18,19].

A particularly important group of wheat–*Thinopyrum* germplasm is the Zhong series of partial amphiploids developed by Sun in China during the early 1980s [20]. Cytogenetic analyses using genomic in situ hybridization (GISH) demonstrated that the Zhong lines contain distinct *Thinopyrum* chromatin segments incorporated into a wheat genomic background [18]. Previous studies further revealed that members of the Zhong series carry valuable resistance to Wheat streak mosaic virus, wheat curl mite, BYDV, wheat stripe rust, and cereal cyst nematodes, highlighting their importance as bridge materials for alien introgression breeding [17,18,19,21]. Nevertheless, resistance to powdery mildew and wheat aphids has not been systematically evaluated in Zhong-derived wheat–*Th. intermedium* advanced lines. More importantly, little is known about whether *Thinopyrum*-derived resistance in these materials can be combined with stable agronomic performance across different genetic backgrounds. The simultaneous improvement of resistance and agronomic performance is particularly important because alien introgressions often exhibit background-dependent phenotypic effects [22,23]. Genetic interactions between introgressed chromatin and recipient wheat genomes may influence resistance expression, plant development, and yield-related traits [22]. Therefore, evaluation of both resistance and agronomic stability is essential for identifying breeding materials with practical value.

In the present study, 159 wheat–*Th. intermedium* derivatives derived from six Zhong partial amphiploids were evaluated for resistance to powdery mildew at both seedling and adult-plant stages, resistance to wheat aphids under field conditions, and key agronomic traits associated with productivity. The objectives were to (i) identify novel sources of resistance to these two major biotic stresses; (ii) determine the frequency of combined resistance; and (iii) assess whether resistance expression is associated with stable agronomic performance across different genetic backgrounds. The results provide insight into the breeding value of Zhong-derived wheat–*Thinopyrum* germplasm and contribute to the development of wheat cultivars combining multi-stress resistance with agronomic stability.

## 2. Results

### 2.1. Parental Background Influences the Frequency and Stability of Powdery Mildew Resistance

**Figure 1:**
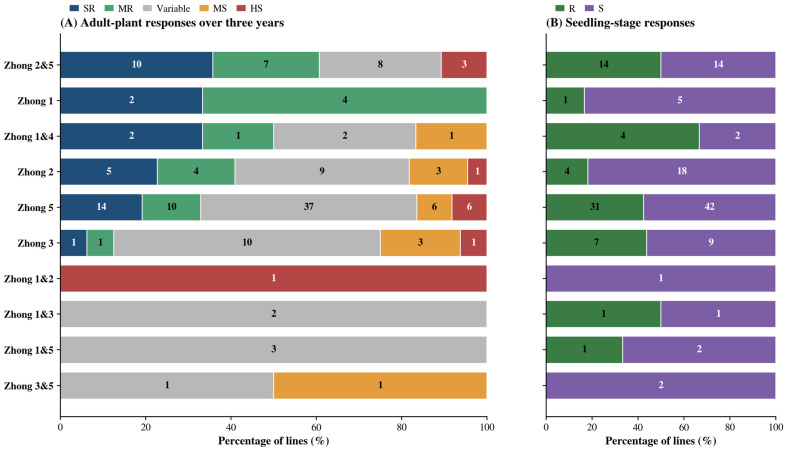
Distribution of powdery mildew responses among wheat–*Th. intermedium* lines from Zhong amphiploids. Lines are grouped by Zhong parent. (**A**) Adult-plant responses over three years: stable resistant (SR), moderately resistant (MR), moderately susceptible (MS), highly susceptible (HS), or variable. (**B**) Seedling-stage responses: resistant (R) or susceptible (S). Bars show percentages within each group; numbers inside bars indicate absolute line counts.

Adult-plant responses were evaluated over three consecutive years (Figure 1A). Among the 159 lines tested, 34 lines (21.4%) exhibited stable resistance (SR) and 27 (17.0%) were moderately resistant (MR). Only 26 lines (16.4%) were susceptible, including 14 moderately susceptible (MS) and 12 highly susceptible (HS) lines, whereas the remaining lines showed variable responses, indicating substantial year-to-year fluctuation in infection phenotype.

The frequency of SR varied among parental backgrounds. Zhong 2 & 5 derivatives had the highest proportion of SR (10/28, 35.7%), followed by Zhong 1 (2/6, 33.3%) and Zhong 1 & 4 (2/6; 33.3%). Intermediate frequencies occurred in Zhong 2 (5/22, 22.7%) and Zhong 5 derivatives (14/73, 19.2%), whereas Zhong 3 yielded only one SR line (1/16, 6.3%). Although Zhong 5 had the most SR lines (n = 14), this reflected its larger population size, not superior resistance frequency. Moderately resistant lines were distributed across most backgrounds, including Zhong 1 (4/6, 66.7%), Zhong 2 (4/22, 18.2%), Zhong 3 (1/16, 6.3%), Zhong 5 (10/73, 13.7%), Zhong 1 & 4 (1/6, 16.7%), as well as Zhong 2 & 5 derivatives (7/28, 25.0%). Variable responses were especially frequent in Zhong 5 (37/73, 50.7%), Zhong 3 (10/16, 62.5%), Zhong 2 (9/22, 40.9%), and Zhong 2 & 5 lines (8/28, 28.6%), indicating substantial year-to-year instability. Susceptibility was unevenly distributed. MS lines occurred mainly in Zhong 2 (3/22, 13.6%), Zhong 3 (3/16, 18.8%), and Zhong 5 (6/73, 8.2%), with single lines in Zhong 1 & 4 (1/6, 16.7%) as well as Zhong 3 & 5 (1/2, 50.0%). HS reactions occurred in Zhong 1 & 2 (1/1, 100%), Zhong 2 (1/22, 4.5%), Zhong 3 (1/16, 6.3%), Zhong 5 (6/73, 8.2%), as well as Zhong 2 & 5 (3/28; 10.7%).

Considered across the full adult-plant response spectrum, clear differences among parental backgrounds were observed. Zhong 5 derivatives (n = 73) displayed the broadest phenotypic distribution, with 14 SR, 10 MR, 37 variable, 6 MS, and 6 HS lines, whereas Zhong 2 & 5 derivatives (n = 28) combined the highest stable resistance proportion with relatively limited susceptibility. Zhong 3 derivatives (n = 16) were dominated by variable responses, with only one SR and one MR line, while the smaller Zhong 1 population (n = 6) contained only SR and MR lines and no susceptible reactions. Thus, parental effects on both resistance frequency and phenotypic stability were clear, with Zhong 2 & 5, as well as Zhong 1 & 4, making the strongest proportional contributions to stable adult-plant resistance.

At the seedling stage (Figure 1B), the frequency of resistant genotypes was lower than that observed for adult-plant resistance. Sixty-three lines (39.6%) were resistant (R) and 96 (60.4%) susceptible (S). The highest resistance frequencies occurred in Zhong 1 & 4 (4/6, 66.7%) and Zhong 2 & 5 derivatives (14/28, 50.0%), followed by Zhong 3 (7/15, 46.7%) and Zhong 5 (31/73, 42.5%). In contrast, resistance was infrequent in Zhong 1 (1/6, 16.7%) and Zhong 2 (4/22, 18.2%). Despite contributing the largest number of resistant seedlings (n = 31), Zhong 5 derivatives also had the most susceptible lines (42/73, 57.5%), again reflecting population size effects.

Across growth stages, resistance was more stable and more frequently expressed at the adult-plant stage than at the seedling stage. The high proportion of variable adult-plant responses, together with increased susceptibility at the seedling stage, suggests that a substantial fraction of lines carry adult-plant or quantitatively expressed resistance rather than effective seedling resistance. Overall, resistance expression was strongly influenced by parental background and developmental stage.

### 2.2. Limited and Unevenly Distributed Resistance to Naturally Occurring Wheat Aphids Among Zhong-Derived Wheat–Th. intermedium Lines

**Figure 2:**
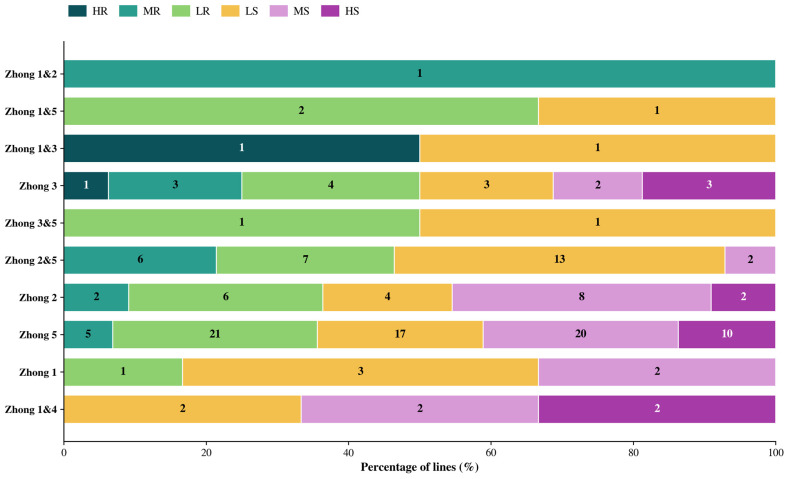
Distribution of responses to wheat aphid under field conditions in wheat–*Th. intermedium* lines derived from the Zhong amphiploids. Lines were grouped by the Zhong parental backgrounds. Aphid damage was assessed using a six-class rating scale: highly resistant (HR), moderately resistant (MR), low resistant (LR), less susceptible (LS), moderately susceptible (MS), and highly susceptible (HS). Stacked bars show the proportional distribution of lines by genotype group; absolute numbers are indicated within the bars.

Field assessment of aphid damage caused by *S. miscanthi* and *S. graminum* revealed generally low levels of resistance in the 159 wheat–*Th. intermedium* advanced lines (Figure 2). Only 61 lines (38.4%) exhibited resistance, including 2 highly resistant (HR; 1.3%), 17 moderately resistant (MR; 10.7%), and 42 low resistant (LR; 26.4%) lines. The remaining 98 lines (61.6%) were susceptible, comprising 45 low susceptible (LS; 28.3%), 36 moderately susceptible (MS; 22.6%), and 17 highly susceptible (HS; 10.7%) lines.

Highly resistant responses were rare, detected in only two lines: one Zhong 3-derived line (1/16) and one Zhong 1 & 3 derivative (1/2). Of 17 moderately resistant lines, the highest frequency was in Zhong 2 & 5 derivatives (6/28, 21.4%), followed by Zhong 5 (5/73, 6.9%), Zhong 3 (3/16, 18.8%), and Zhong 2 derivatives (2/22, 9.1%), with one from Zhong 1 & 2 (1/1). Low resistance constituted the largest resistant class (42 lines), predominantly from Zhong 5 derivatives (21/73, 28.8%), followed by Zhong 2 & 5 (7/28, 25.0%), Zhong 2 (6/22, 27.3%), and Zhong 3 (4/16, 25.0%) backgrounds. Smaller contributions came from Zhong 1 & 5 (2/3, 66.7%), Zhong 1 (1/6), and Zhong 3 & 5 derivatives (1/2).

Susceptible responses predominated across most parental backgrounds. Within the LS category, 45 lines were identified, including 17 Zhong 5 derivatives (17/73, 23.3%), and 13 Zhong 2 & 5 derivatives (13/28; 46.4%), and smaller numbers from Zhong 2 (4/22, 18.2%), Zhong 1 (3/6), Zhong 3 (3/16, 18.8%), Zhong 1 & 4 (2/6, 33.3%), and single lines from Zhong 1 & 3, Zhong 1 & 5, and Zhong 3 & 5 backgrounds. The 36 moderately susceptible lines were mainly from Zhong 5 (20/73, 27.4%) and Zhong 2 (8/22, 36.4%), with two lines each from Zhong 1, Zhong 3, Zhong 1 & 4, and Zhong 2 & 5 backgrounds. Seventeen lines showed highly susceptible reactions, including ten Zhong 5 derivatives (10/73, 13.7%), three Zhong 3 derivatives (3/16, 18.8%), and two lines each from Zhong 2 (2/22, 9.1%) and Zhong 1 & 4 (2/6, 33.3%).

Across parental backgrounds, the distribution of resistance categories differed substantially. Zhong 5 derivatives (n = 73) contributed the most lines to both resistant (5 MR and 21 LR) and susceptible classes (17 LS, 20 MS, and 10 HS), reflecting their larger sample size. Zhong 2 & 5 derivatives (n = 28) showed a relatively higher frequency of resistance, including six MR and seven LR lines, although 17 lines remained susceptible. Zhong 3 derivatives (n = 16) included the only HR line among the major parental groups and displayed a broad range of responses across all categories. In contrast, Zhong 2 derivatives (n = 22) were predominantly susceptible, with 14 lines classified as MS or HS. Minor parental groups showed limited representation but variable responses, including the HR line from Zhong 1 & 3 and an MR line from Zhong 1 & 2.

### 2.3. Uneven Distribution of Single, Dual, and Triple Resistance Across Zhong-Derived Genetic Backgrounds

**Figure 3:**
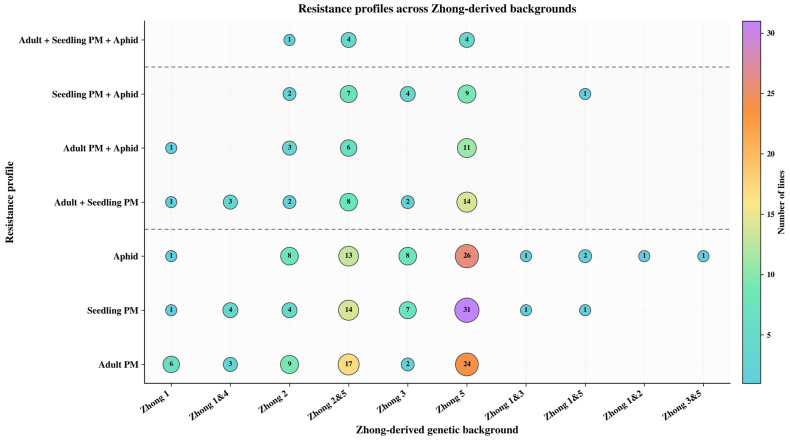
Distribution of resistance profiles across Zhong-derived genetic backgrounds. Bubble plot showing the distribution of wheat–*Th. intermedium* advanced breeding lines with resistance to adult-stage powdery mildew (Adult PM), seedling-stage powdery mildew (Seedling PM), and aphids, either individually or in combination. Resistance profiles were grouped into single resistance (Adult PM, Seedling PM, or Aphid), dual resistance (Adult PM + Seedling PM, Adult PM + Aphid, or Seedling PM + Aphid), and triple resistance (Adult PM + Seedling PM + Aphid). Genetic backgrounds correspond to lines derived from different Zhong amphiploid parents or their combinations. Bubble size is proportional to the number of lines in each category, with exact counts indicated within the circles. Horizontal dotted lines separate the single-, dual-, and triple-resistance classes.

The distribution of resistance traits differed substantially among Zhong-derived genetic backgrounds, particularly in the occurrence of single and combined resistance to powdery mildew and aphids (Figure 3). Resistance to individual stresses was relatively common across the evaluated germplasm. Adult-plant stage powdery mildew resistance was found in several backgrounds, most frequently in Zhong 5 (24 lines) and Zhong 2 & 5 derivatives (17 lines), followed by Zhong 2 (9 lines) and Zhong 1 (6 lines), with smaller contributions from Zhong 1 & 4 (3 lines) and Zhong 3 (2 lines). A similar trend was observed for seedling resistance to powdery mildew, with Zhong 5 derivatives again having the most lines (31), followed by Zhong 2 & 5 (14 lines), Zhong 3 (7 lines), Zhong 2 (4 lines), and Zhong 1 & 4 (4 lines), whereas Zhong 1 contributed only one line. Aphid resistance was also broadly distributed, again dominated by Zhong 5 derivatives (26 lines), followed by Zhong 2 & 5 (13 lines), Zhong 2 and Zhong 3 (eight each), with isolated cases in Zhong 1, Zhong 1 & 3, Zhong 1 & 2, and Zhong 3 & 5 backgrounds.

In contrast to single resistance, combined resistance traits were less frequent and showed a more restricted distribution among parental backgrounds. Dual resistance to powdery mildew at both developmental stages was observed in 32 lines, primarily within Zhong 5 (14 lines) and Zhong 2 & 5 (8 lines) derivatives, with smaller contributions from Zhong 1 & 4 (3 lines), Zhong 2 (2 lines), Zhong 3 (2 lines), and Zhong 1 (1 line). Resistance combining adult-plant powdery mildew resistance (APR) to powdery mildew and aphids occurred in 21 lines, including 11 Zhong 5 derivatives, six Zhong 2 & 5 derivatives, three Zhong 2 derivatives, and one Zhong 1-derived line. Similarly, seedling resistance to powdery mildew and aphid resistance were found in 23 lines, most frequently among Zhong 5 (9 lines) as well as Zhong 2 & 5 (7 lines) backgrounds, followed by Zhong 3 (4 lines), Zhong 2 (2 lines), and Zhong 1 & 5 (1 line). Triple resistance to adult-plant stage powdery mildew, seedling-stage powdery mildew, and aphids was rare, detected in only nine lines overall (Table S1). These lines were confined to a limited number of genetic backgrounds, including four Zhong 5 derivatives, four Zhong 2 & 5 derivatives, and a single Zhong 2-derived line.

### 2.4. Effects of Year, Pedigree Background, and Resistance Classes on Agronomic Traits

Analysis of variance (ANOVA) revealed significant effects of year on all agronomic traits evaluated in both the powdery mildew and aphid resistance models (Table 1), indicating a strong environmental influence on trait expression. In the powdery mildew model, year significantly affected plant height, spike length, spikelet number per spike, and grain number per spike (*p* < 0.01 or *p* < 0.001), while pedigree background significantly influenced plant height (*p* < 0.01). In the aphid model, year significantly affected all four agronomic traits, and pedigree background had a significant effect on plant height (*p* < 0.05). Neither the APR class nor the aphid response class showed significant main effects on plant height, spike length, spikelet number per spike, or grain number per spike. These results indicate that differences in resistance level were not associated with substantial changes in agronomic performance among the evaluated Zhong-derived wheat–*Th. intermedium* materials.

Several significant interaction effects were detected. In the powdery mildew model, year × pedigree interactions significantly affected plant height, spikelet number per spike, and grain number per spike, whereas year × APR and pedigree × APR interactions were significant only for grain number per spike. In the aphid model, year × pedigree, year × aphid response, and pedigree × aphid response interactions significantly influenced selected traits, particularly spikelet number per spike. However, no significant three-way interactions were detected in either model.

Overall, environmental variation exerted a substantially greater influence on agronomic trait expression than either powdery mildew or aphid resistance level, while moderate genotype-by-environment interactions contributed to variation in several yield-related traits.

### 2.5. Variance Components of Agronomic Traits Across Environments

**Figure 4:**
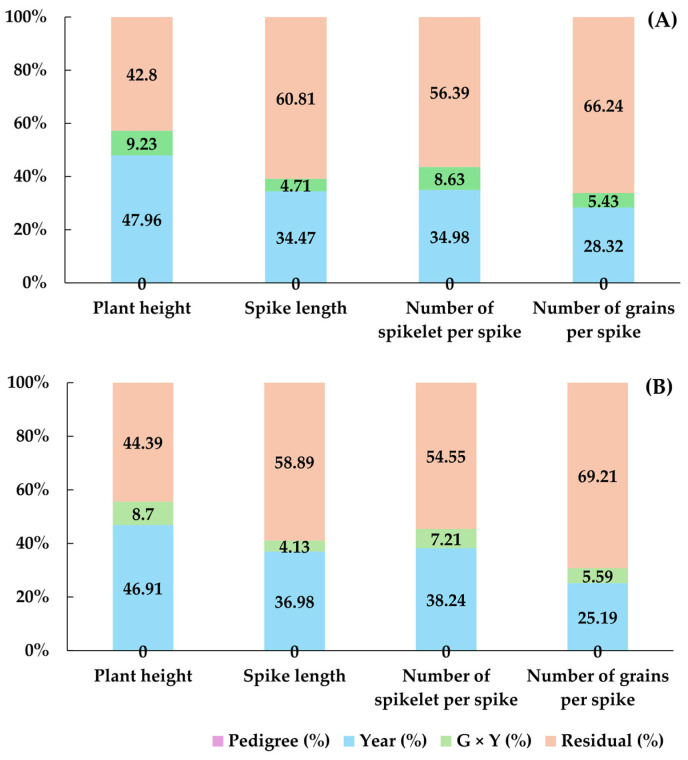
Relative contributions of variance components to agronomic trait variation in Zhong-derived wheat–*Th. intermedium* materials. (**A**) Powdery mildew model; (**B**) Aphid model. Variance components were estimated using a mixed-effects model. Bars represent the proportion of total phenotypic variance attributable to pedigree background, year, genotype × year interaction (G × Y), and residual variance for plant height, spike length, spikelet number per spike, and grain number per spike.

Variance component analysis revealed broadly similar patterns in the powdery mildew and aphid resistance models (Figure 4). Across all agronomic traits, year accounted for the largest proportion of phenotypic variation, explaining 25.19–46.91% of the variance in the powdery mildew model and 28.32–47.96% in the aphid model. The contribution of the year was greatest for plant height, accounting for 46.91% and 47.96% of the total variance in the powdery mildew and aphid models, respectively.

Genotype × year (G × Y) interactions contributed a relatively small proportion of the total variance, ranging from 4.13% to 8.70% in the powdery mildew model and from 4.71% to 9.23% in the aphid model. The largest G × Y contribution was observed for plant height in both analyses.

Residual variance represented the largest component for spike length, spikelet number per spike, and grain number per spike, accounting for 54.55–69.21% of the total variance in the powdery mildew model and 56.39–66.24% in the aphid model. In contrast, pedigree effects contributed negligibly to the total variance for all traits.

Together with the ANOVA results, these findings indicate that environmental variation was the primary determinant of agronomic trait expression in the Zhong-derived materials, whereas genotype × environment interactions contributed modestly and pedigree effects were comparatively small.

Although pedigree background had no significant effect on spike length, spikelet number per spike, or grain number per spike, significant differences were detected for plant height in both the powdery mildew and aphid resistance models (Table 1). Multiple comparisons revealed moderate variation in plant height among Zhong-derived pedigree groups (Figure S1). Across the evaluated materials, mean plant height ranged from 70.8 to 115.3 cm, with taller plants generally observed in materials derived from Zhong 2 and Zhong 3 backgrounds, whereas several Zhong 1 and Zhong 4 derivatives exhibited comparatively shorter stature. Nevertheless, the observed differences among pedigree groups were modest and showed no consistent association with powdery mildew or aphid resistance responses.

### 2.6. Association of Agronomic Traits with Powdery Mildew and Aphid Resistance

**Figure 5:**
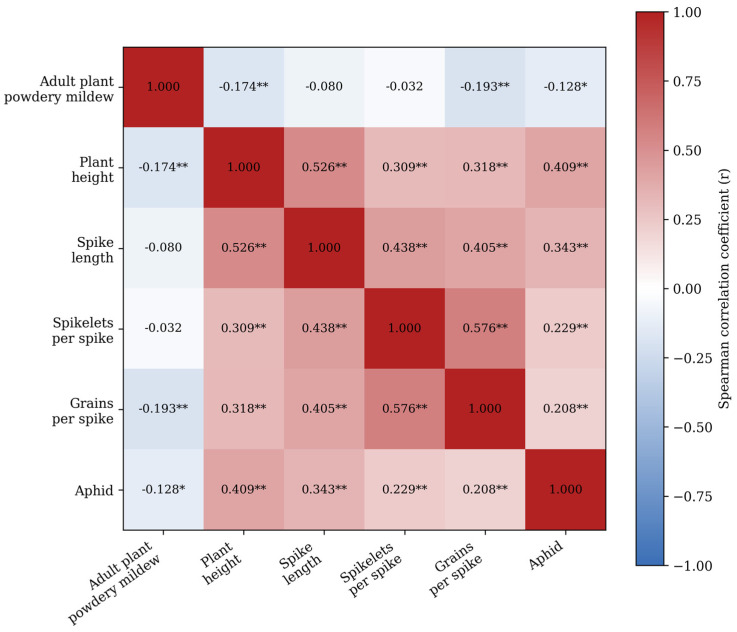
Spearman rank correlation analysis among adult-plant powdery mildew response, aphid resistance, and agronomic traits in Zhong-derived wheat–*Thinopyrum intermedium* materials. Heatmap showing pairwise Spearman correlation coefficients (*r_s_*) among adult-plant powdery mildew response, aphid resistance, plant height, spike length, number of spikelets per spike, and number of grains per spike. Color intensity represents the strength and direction of the correlation, with blue indicating negative correlations and red indicating positive correlations. Spearman correlation coefficients (*r_s_*) are displayed within cells. Asterisks indicate significant correlations (* *p* < 0.05; ** *p* < 0.01, two-tailed test).

Spearman rank correlation analysis revealed generally weak associations between disease resistance and agronomic traits among the evaluated Zhong-derived wheat–*Th. intermedium* materials (Figure 5). Adult-plant powdery mildew response was negatively correlated with plant height (*r_s_* = −0.174, *p* = 0.002) and grain number per spike (*r_s_* = −0.193, *p* = 0.001), whereas no significant correlations were detected with spike length or spikelet number per spike. These relationships suggest that lines with improved powdery mildew resistance tended to maintain slightly greater plant height and grain number per spike.

Aphid response was negatively correlated with powdery mildew response (*r_s_* = −0.128, *p* = 0.025) and positively correlated with plant height (*r_s_* = 0.409, *p* < 0.001), spike length (*r_s_* = 0.343, *p* < 0.001), spikelet number per spike (*r_s_* = 0.229, *p* < 0.001), and grain number per spike (*r_s_* = 0.208, *p* < 0.001).

Among agronomic traits, grain number per spike was positively correlated with spikelet number per spike (*r_s_* = 0.576, *p* < 0.001), spike length (*r_s_* = 0.405, *p* < 0.001), and plant height (*r_s_* = 0.318, *p* < 0.001). Plant height was also positively associated with spike length (*r_s_* = 0.526, *p* < 0.001) and spikelet number per spike (*r_s_* = 0.309, *p* < 0.001).

## 3. Discussion

### 3.1. Zhong-Derived Wheat–Th. intermedium Materials Represent Valuable Sources of Adult-Plant Powdery Mildew Resistance

Powdery mildew, caused by Bgt, remains one of the most widespread foliar diseases of wheat and continues to threaten grain production in many wheat-growing regions worldwide. Although numerous powdery mildew resistance genes have been deployed in wheat breeding, the effectiveness of many race-specific resistance genes has declined due to the rapid evolution of pathogen populations [9,24]. Consequently, the identification of new and potentially durable sources of resistance remains a major objective in wheat improvement programs [25].

APR is generally considered more durable than seedling resistance because it is often quantitatively inherited and less vulnerable to rapid breakdown by changes in pathogen virulence [9]. Wild relatives of wheat have contributed substantially to the enrichment of the wheat resistance gene pool, and *Th. intermedium* has been recognized as an important source of resistance to multiple diseases, including powdery mildew, stripe rust, leaf rust, and Fusarium head blight [13]. Several resistance genes and chromosome segments originating from *Thinopyrum* species have been successfully transferred into wheat, demonstrating the value of these species for broadening genetic diversity and improving disease resistance [13].

The present study identified substantial variation in adult-plant powdery mildew response among the Zhong-derived wheat–*Th. intermedium* materials. Importantly, resistant phenotypes were observed repeatedly across three consecutive growing seasons, despite variation in disease pressure among years. While the genetic basis of resistance remains unknown, the consistent performance of several lines under field conditions suggests that these materials represent useful sources of APR for further genetic characterization and breeding utilization.

It is noteworthy that the Zhong amphiploids were originally developed to facilitate the transfer of favorable traits from *Th. intermedium* into wheat backgrounds. Previous studies involving wheat–*Thinopyrum* introgressions have primarily focused on the identification of novel resistance genes or chromosome substitutions and translocations [26,27]. In contrast, the present work evaluated a large collection of derived materials under field conditions and demonstrated the presence of stable phenotypic variation for powdery mildew response within this germplasm. These findings provide a foundation for future cytogenetic and molecular analyses aimed at identifying the chromosomal regions associated with resistance and determining their potential value in wheat improvement programs.

### 3.2. Aphid Resistance Identified in Zhong-Derived Materials Expands the Utility of Thinopyrum-Derived Germplasm

Aphids are among the most important insect pests of wheat worldwide because they cause direct feeding damage and serve as vectors of economically significant viral diseases. Yield losses resulting from aphid infestation can be substantial, particularly under favorable environmental conditions that support rapid population growth [28]. Although insecticide application remains an important component of aphid management, increasing concerns regarding production costs, environmental impacts, and the evolution of insecticide resistance have highlighted the need for host-plant resistance as a sustainable control strategy [29,30].

Compared with resistance to fungal diseases, effective sources of aphid resistance are relatively uncommon in bread wheat. Previous studies have shown that the genetic diversity available within cultivated wheat provides only limited resistance to several economically important aphid species, prompting extensive efforts to identify novel resistance sources from wild relatives and related species [30,31]. Alien introgressions from species such as rye (*Secale cereale*), *Aegilops* spp., and *Thinopyrum* spp. have therefore attracted considerable interest as potential sources of insect resistance for wheat improvement [29].

In the present study, substantial variation in aphid response was observed among the Zhong-derived wheat–*Th. intermedium* materials, with several lines consistently exhibiting lower levels of infestation under natural field conditions. Although the current evaluation was based on visual scoring rather than quantitative measurements of aphid abundance, the results indicate that useful variation for aphid response exists within this germplasm. The identification of resistant materials is particularly noteworthy because most previous studies involving wheat–*Thinopyrum* introgressions have focused primarily on resistance to fungal diseases, whereas insect resistance has received comparatively less attention.

The observed variation may reflect the contribution of *Thinopyrum*-derived chromatin, although the underlying genetic basis remains unknown. Resistance to aphids can involve multiple mechanisms, including antibiosis, antixenosis, and tolerance, which frequently show quantitative inheritance and strong environmental influences [29,32]. Consequently, further studies incorporating aphid population counts, temporal monitoring of infestation dynamics, and characterization of resistance mechanisms will be necessary to confirm and refine the phenotypic assessments reported here.

Despite these limitations, the present results broaden the range of potentially useful traits identified in Zhong-derived wheat–*Th. intermedium* materials. The occurrence of aphid-resistant lines exhibiting favorable powdery mildew responses highlights the value of this germplasm as a resource for future resistance breeding and genetic studies.

It should be noted that the aphid evaluation was conducted under natural field infestation conditions and was based on visual assessment of plant response. Aphid abundance, species composition, and temporal population dynamics were not quantified. Therefore, the aphid response classes reported here should be regarded as preliminary field-based assessments requiring validation through quantitative monitoring and controlled infestation experiments.

### 3.3. Resistance and Agronomic Performance in Zhong-Derived Wheat–Th. intermedium Materials

The relationship between resistance and agronomic performance remains an important consideration in the utilization of alien introgressions for wheat improvement. Resistance loci transferred from wild relatives may be associated with unfavorable agronomic effects through linkage drag or pleiotropic interactions, potentially limiting their breeding value [26,27]. Consequently, the identification of resistant materials that maintain acceptable agronomic performance is a major objective in pre-breeding programs [33,34].

The statistical analyses conducted in the present study provided limited evidence for strong associations between resistance responses and agronomic traits. Neither adult-plant powdery mildew resistance class nor aphid response class exerted significant main effects on plant height, spike length, spikelet number per spike, or grain number per spike in the ANOVA. Mixed-model analyses further indicated that year accounted for a substantially larger proportion of phenotypic variation than pedigree background or genotype × year interaction, highlighting the predominant influence of environmental factors on trait expression. Correlation analyses supported these findings. Powdery mildew response showed weak negative associations with plant height and grain number per spike, whereas aphid response exhibited weak to moderate correlations with several agronomic traits. Although some of these relationships were statistically significant, the corresponding correlation coefficients were low, indicating that resistance explained only a small proportion of the observed phenotypic variation. Therefore, resistance status was not a major determinant of agronomic performance within the evaluated population. Variance component analysis provided a similar interpretation. Despite significant differences in plant height among certain pedigree groups, pedigree contributed little to the overall phenotypic variance relative to year and residual effects. These results suggest that agronomic performance was influenced more strongly by environmental conditions than by resistance class or pedigree background.

It should be noted that the present study was designed to evaluate resistance responses rather than tolerance. Because pathogen severity, aphid abundance, and associated damage were not quantified relative to non-infected controls. Consequently, the absence of consistent differences in the agronomic traits evaluated among resistance classes should not be interpreted as evidence of tolerance to powdery mildew or aphid infestation. Further studies incorporating quantitative measurements of disease and pest pressure together with direct assessments of yield loss will be required to address this question.

### 3.4. Agronomic Consequences of Alien Introgressions in Zhong-Derived Wheat–Thinopyrum Backgrounds

The limited contribution of resistance class to agronomic variation may reflect the breeding history of the Zhong-derived materials. Unlike primary alien introgression plant materials, which often contain large chromosomal segments and substantial amounts of non-adapted chromatin, the materials evaluated here have undergone multiple generations of selection within wheat backgrounds [33,34]. Such selection is expected to eliminate highly deleterious introgressions while retaining chromosome segments carrying favorable resistance alleles [26,35]. Consequently, the weak associations observed between resistance responses and agronomic traits may indicate that much of the unfavorable linkage drag commonly associated with alien introgressions has already been reduced during germplasm development.

The negligible contribution of pedigree background to overall phenotypic variance was also noteworthy. The Zhong amphiploids differ in their chromosomal constitution [18] and have served as distinct bridges for transferring *Th. intermedium* chromatin into wheat. Nevertheless, pedigree effects explained little of the total variance for the agronomic traits evaluated. This result suggests that the expression of agronomic performance was largely determined by environmental conditions and within-pedigree variation rather than by the particular Zhong-derived background from which a line originated.

It is also noteworthy that powdery mildew resistance and aphid resistance exhibited contrasting relationships with agronomic performance. Powdery mildew resistance showed only weak associations with plant height and grain number per spike, whereas aphid resistance displayed somewhat stronger correlations with several agronomic traits. This difference may reflect the distinct biological mechanisms underlying pathogen and insect resistance. Disease resistance genes often act through pathogen recognition and defense signaling pathways, whereas resistance to insect pests may involve additional morphological, physiological, or metabolic traits that can influence plant growth and resource allocation [9,29]. Nevertheless, the relatively low correlation coefficients observed in the present study indicate that any trade-offs associated with aphid resistance were modest and unlikely to be a major constraint in breeding.

### 3.5. Breeding Implications of Thinopyrum-Derived Introgressions

The exploitation of genetic variation from wild relatives has become an essential strategy for broadening the genetic base of modern wheat and introducing novel sources of resistance to biotic and abiotic stresses. Among wheat relatives, species of the genus *Thinopyrum* have contributed numerous resistance genes and chromosome segments that have been widely utilized in wheat improvement programs [13,26,33]. However, the deployment of alien chromatin in breeding has historically been constrained by linkage drag, incomplete compensation between wheat and alien chromosomes, and agronomic instability associated with large introgressed segments [26,27]. Consequently, the development of introgression materials that combine resistance with acceptable agronomic performance remains a central objective of wheat pre-breeding.

The Zhong-derived wheat–*Th. intermedium* materials evaluated in the present study provide an opportunity to assess the breeding value of advanced introgression germplasm following multiple generations of selection in wheat backgrounds. In contrast to many early-generation alien introgression lines, where unfavorable agronomic effects are frequently observed, the present analyses revealed limited evidence that resistance responses were strongly associated with reductions in agronomic performance. Although environmental effects accounted for a substantial proportion of the observed variation, neither powdery mildew resistance nor aphid response emerged as major determinants of agronomic trait expression. These observations suggest that at least some of the resistance-carrying chromatin present in the evaluated materials may have been incorporated into wheat backgrounds without the pronounced agronomic penalties often associated with large alien chromosome segments.

The Zhong-derived wheat–*Th. intermedium* materials evaluated in the present study provide an opportunity to assess the breeding value of advanced introgression germplasm developed from distinct Zhong amphiploid backgrounds. In contrast to many early-generation alien introgression lines, where linkage drag and unfavorable agronomic effects are frequently reported [26,27], the present analyses revealed limited evidence that powdery mildew resistance or aphid response was strongly associated with variation in the agronomic traits evaluated. Environmental effects accounted for a substantial proportion of the observed phenotypic variation, whereas resistance classes contributed comparatively little. These findings suggest that the resistance-associated chromatin present in the evaluated materials is not strongly linked to adverse agronomic effects and that favorable combinations of resistance and agronomic performance can be recovered within Zhong-derived wheat–*Thinopyrum* backgrounds.

Previous studies have demonstrated that the breeding value of alien introgressions depends largely on the size and chromosomal location of the transferred segments. Large chromosome additions, substitutions, and translocations frequently carry both favorable and unfavorable alleles, resulting in linkage drag and reduced adaptation [26,27,33]. In contrast, chromosome engineering approaches that reduce introgression size through homoeologous recombination can substantially improve the agronomic performance of resistance-carrying lines while retaining target loci [4,27]. The generally weak associations between resistance and agronomic traits observed here are consistent with the possibility that recombination and selection have already reduced some of the unfavorable effects typically associated with alien chromatin.

The results also highlight the importance of integrating modern cytogenetic and genomic tools into future utilization of Zhong-derived germplasm. Precise characterization of introgressed chromosome segments using molecular markers, genomic in situ hybridization, high-density SNP platforms, and whole-genome genotyping will facilitate identification of the chromosomal regions underlying resistance and enable further refinement of introgression size [12,33]. Such approaches have become increasingly important in wheat chromosome engineering, allowing breeders to retain beneficial alleles while minimizing residual linkage drag.

Although the resistance mechanisms and chromosomal constitution of the evaluated materials remain to be fully characterized, the present study identified lines combining favorable powdery mildew responses, reduced aphid infestation, and acceptable agronomic performance under field conditions. These materials, therefore, represent valuable pre-breeding resources for the development of improved wheat germplasm and provide a foundation for future genetic dissection, chromosome engineering, and marker-assisted introgression of resistance-associated chromatin.

## 4. Materials and Methods

### 4.1. Plant Materials

One hundred fifty-nine advanced wheat–*Th. intermedium* derivatives, developed from the Zhong series comprising four sister partial amphiploid lines (Zhong 1, Zhong 2, Zhong 3, and Zhong 5), were included. Derivatives were grouped by their respective Zhong parent based on pedigree. The Zhong series was developed in the early 1980s by hybridizing common wheat cultivars and a *Th. intermedium* accession introduced from the former USSR. The wheat parents included the Chinese spring cultivars Hezuo 2, Daqingmang, and Keqiang, and the Italian spring cultivar Mentana [36].

### 4.2. Assessment of Adult-Plant Resistance to Powdery Mildew

Adult-plant resistance (APR) to powdery mildew was evaluated under field conditions during three consecutive growing seasons (2023–2025) at the experimental station of Shanxi Agricultural University, Dongyang, Shanxi Province, China (37°55′ N, 112°67′ E). Field trials were conducted using a randomized complete block design with three biological replicates each year. Each wheat line was planted in a 1.5 m row with 0.3 m spacing between rows, and approximately 50 seeds were sown per row in early October each year. To minimize positional effects, plots within each block were randomized annually. The susceptible wheat cultivar Zhongzuo 9504 was planted as a spreader row after every 10 test rows to ensure uniform disease distribution. Standard agronomic management practices for local wheat production were applied, except that no fungicides or insecticides were used during the experimental period. Disease pressure was established by artificial inoculation in early April at the jointing stage [37]. A mixed population of Bgt isolates, including E09, E15, E21, E23-(2), and E31, was used for inoculation [38]. These isolates were maintained on susceptible wheat seedlings under controlled growth chamber conditions. After inoculation, supplementary irrigation was applied when necessary to maintain favorable humidity for disease development. Disease assessments were conducted in early June at the early-to-mid grain filling stages [37], when the susceptible control exhibited severe symptoms. Five plants per plot were evaluated weekly, with at least two independent observations per plant. Infection type (IT) at late grain filling (GS90) was scored using a 0–9 scale: 0, no infection; 1, limited lesions on the lowest leaves; 3, slight infection on the lower third of the plant; 5, severe infection on lower leaves with moderate spread to middle leaves; 7, severe infection on lower and middle leaves with moderate infection on the flag leaf; 9, severe infection across all leaves and spike. Intermediate scores (2, 4, 6, 8) were used for finer discrimination. IT ratings were classified into five categories: immunity (I, 0), high resistance (HR, 1–2), moderate resistance (MR, 3–4), moderate susceptibility (MS, 5–6), and high susceptibility (HS, 7–9) [39].

Across three consecutive years, consensus adult-plant infection types were assigned according to the multi-year response pattern. Lines with consistently resistant reactions (e.g., I/HR/MR, I/I/MR, and HR/HR/MR) were classified as stable resistant (SR); those with predominantly moderate resistance (e.g., MR/MR/MR, HR/MR/MR, and I/MR/MR) as moderately resistant (MR); those showing inconsistent reactions across years as variable (V); those with moderate susceptibility (e.g., MS/MS/MS and MS/MS/HS) as moderately susceptible (MS); and those with persistent high suscepti-bility (e.g., MS/HS/HS and HS/HS/HS) as highly susceptible (HS).

### 4.3. Assessment of Resistance to Powdery Mildew at the Seedling Stage

Seedling-stage resistance to powdery mildew was evaluated in a greenhouse under controlled conditions with a 14 h light/10 h dark photoperiod at 22 °C/18 °C (day/night). The experiment followed a completely randomized design with three biological replicates. Six seeds per line were planted in plastic trays (54 × 28 × 4.2 cm) containing 128 wells (3.2 × 3.2 × 4.2 cm). The susceptible cultivar Zhongzuo 9504 was included in each tray. At the one-leaf stage, seedlings were inoculated with fresh conidia of Bgt isolate Bgt27 [37]. Approximately 10 mg of conidia per tray were evenly dusted onto seedlings to ensure uniform infection. Following inoculation, seedlings were maintained at high relative humidity (>70%) to promote disease development. Two weeks post-inoculation, when pustules were fully developed on the first leaf of the susceptible control, IT was assessed for each plant on a 0–4 scale: 0, no visible infection; 0;, hypersensitive flecks without sporulation; 1, restricted sporulation with necrosis and chlorosis; 2, moderate sporulation with necrotic and chlorotic areas; 3, sporulation with chlorosis, and 4, abundant sporulation without chlorosis. Based on the IT ratings, host responses were classified as resistant (IT 0–2) or susceptible (IT 3–4) [40].

### 4.4. Evaluation of Visual Damage Rating Caused by Natural Field Infection of Wheat Aphids

Field resistance to aphids was evaluated under natural infestation conditions during the 2024 and 2025 growing seasons at the same experimental location. Trials were arranged using a randomized complete block design with three replicates. Each wheat line was planted in a 1.5 m row with 0.3 m spacing between rows, and approximately 50 seeds were sown per row in early October each year. No insecticides were applied during the experiment to allow natural aphid population development. Aphid populations consisted predominantly of *Sitobion miscanthi* and *Schizaphis graminum*. Aphid infestation occurred naturally from late April to early June, corresponding to heading and grain filling stages. Disease and pest pressure were considered moderate to high during both seasons based on the performance of the susceptible control. Visual damage ratings were conducted at peak aphid infestation using the 1–6 scale described by Scott et al. [41]. Ten plants per plot were evaluated, and the mean score was used for classification. The scale was 1-6, where: 1, no damage or small, isolated chlorotic spots, high resistance (HR); 2, large, isolated chlorotic spots, moderate resistance (MR); 3, chlorotic spots merging into a few large chlorotic patches, low resistance (LR); 4, numerous and prominent chlorotic patches with pale yellow or white streaking, less susceptibility (LS); 5, severe chlorosis and/or prominent yellow or white streaking, with some wilting, moderate susceptibility (MS); 6, plants dead or dying, severe wilting, with most plants flat on the ground and aphids migrating to other plants, high susceptibility (HS).

### 4.5. Evaluation of Agronomic Traits

In two consecutive growing seasons (2023–2024 and 2024–2025), spike length was measured from the rachis base to the spike tip. Plant height was measured at maturity (GS90) from ground level to the spike tip, excluding awns. The number of spikelets per spike was counted. Ten randomly selected plants per plot were measured for each trait. For grain number, spikes were threshed manually with a bench micro-thresher.

### 4.6. Statistical Analysis

All statistical analyses were conducted using Jamovi v2.7.31 (The Jamovi Project, Sydney, Australia) [42]. Agronomic traits were analyzed separately for the powdery mildew and aphid evaluation datasets.

Analysis of variance (ANOVA) was performed to assess the effects of year, pedigree background, resistance response class, and their interactions on agronomic traits. For the powdery mildew dataset, the model included year, pedigree, APR class, and their interactions. For the aphid dataset, year, pedigree, aphid response class, and their interactions were included in the model. Mean separation was conducted using Fisher’s least significant difference (LSD) test at the 0.05 probability level when significant main effects were detected.

To quantify the relative contributions of different sources of variation, mixed-model analyses were performed with year, pedigree, and genotype × year interaction treated as random effects. Variance components were estimated for each agronomic trait and expressed as percentages of the total phenotypic variance.

Associations between disease response and agronomic traits were evaluated using Spearman’s rank correlation coefficients. Correlation analyses were conducted between powdery mildew response, aphid response, plant height, spike length, spikelet number per spike, and grain number per spike. Correlation significance was assessed at *p* < 0.05.

## 5. Conclusions

Zhong-derived wheat–*Th*. *intermedium* materials represent a valuable source of resistance to powdery mildew and aphids in wheat. Several lines exhibited favorable resistance responses across years, although combined resistance to both stresses was uncommon. Agronomic variation was influenced primarily by environmental effects, while pedigree background and resistance responses contributed comparatively little to overall phenotypic variation. No strong associations were detected between resistance responses and agronomic traits, suggesting that major agronomic penalties were not evident within the evaluated materials. These findings highlight the potential of Zhong-derived wheat–*Thinopyrum* germplasm as a useful resource for pre-breeding and for the development of wheat cultivars with improved resilience to multiple biotic stresses.

## Figures and Tables

**Table 1:** Summary of ANOVA results for agronomic traits of Zhong-derived wheat–*Th. intermedium* materials.

Source of Variation	df	Plant Height	Spike Length	Spikelets per Spike	Grain Number per Spike
Powdery mildew model					
Year	1	***	***	***	**
Pedigree	9	**	ns	ns	ns
APR class	4	ns	ns	ns	ns
Year × Pedigree	8	*	ns	***	*
Year × APR	4	ns	ns	ns	*
Pedigree × APR	17	ns	ns	ns	*
Year × Pedigree × APR	9	ns	ns	ns	ns
Aphid model					
Year	1	***	*	***	***
Pedigree	9	*	ns	ns	ns
Aphid response	6	ns	ns	ns	ns
Year × Pedigree	4	*	ns	**	ns
Year × Aphid	4	ns	ns	***	ns
Pedigree × Aphid	26	ns	ns	*	ns
Year × Pedigree × APR	6	ns	ns	ns	ns

ns = not significant, * *p* < 0.05, ** *p* < 0.01, *** *p* < 0.001.

## Data Availability

The data presented in this study are available in Supplementary Dataset S1 accompanying this article. The dataset includes the phenotypic evaluations of the wheat–*Th*. *intermedium* derivatives used in the present study. Additional information may be obtained from the corresponding author upon reasonable request.

## Supplementary Materials

The following supporting information can be downloaded at: https://www.mdpi.com/article/10.3390/plants15121894/s1.
