# Supplementary material for: Powdery Mildew and Aphid Resistance in Wheat–Thinopyrum intermedium Derivatives from Zhong Backgrounds
Source: Plants (Basel). 2026 Jun 18;15(12):1894. doi: 10.3390/plants15121894 (PMC13306772; doi:10.3390/plants15121894)
Supplement: Supplementary file 1 [file plants-15-01894-s001.zip › Figure S1.pdf]

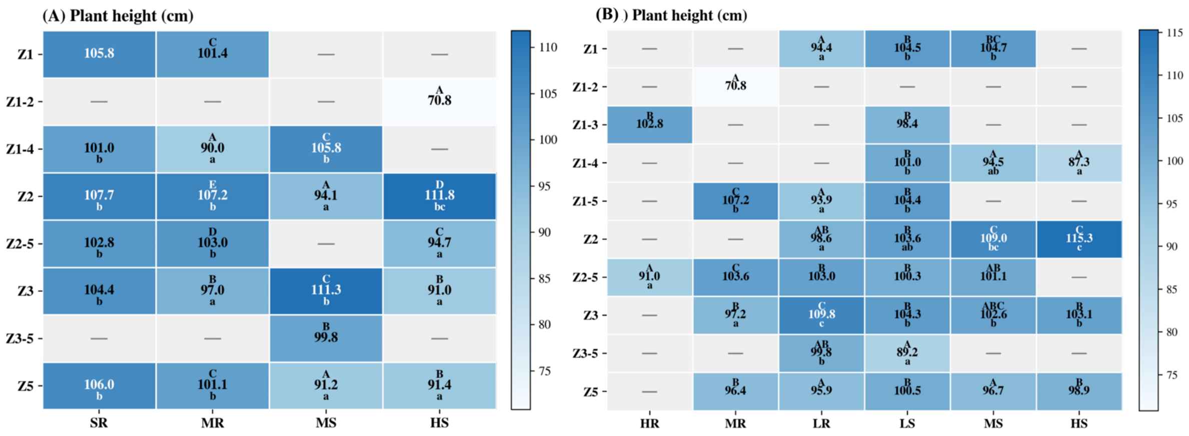

Figure S1. Plant height of Zhong-derived wheat—*Th. intermedium* advanced lines under different adult-plant stage powdery mildew infection responses (A) and aphid responses (B). Lines were grouped by pedigree: Zhong 1 (Z1), Zhong 1 and 2 (Z1–2), Zhong 1 and 4 (Z1–4), Zhong 2 (Z2), Zhong 2 and 5 (Z2–5), Zhong 3 (Z3), Zhong 3 and 5 (Z3–5), and Zhong 5 (Z5). Adult-plant stage powdery mildew infection types were stable resistant (SR), moderately resistant (MR), moderately susceptible (MS), or highly susceptible (HS). Aphid responses were classified as highly resistant (HR), moderately resistant (MR), low resistant (LR), low susceptible (LS), moderately susceptible (MS), or highly susceptible (HS). Capital letters above mean values indicate significant differences among pedigrees within the same infection type, whereas lowercase letters below mean values indicate significant differences among infection types within the same pedigree ($p < 0.05$). Absence of letters denotes no significant difference.
