# Supplementary material for: Powdery Mildew and Aphid Resistance in Wheat–Thinopyrum intermedium Derivatives from Zhong Backgrounds
Source: Plants (Basel). 2026 Jun 18;15(12):1894. doi: 10.3390/plants15121894 (PMC13306772; doi:10.3390/plants15121894)
Supplement: Supplementary file 1 [file plants-15-01894-s001.zip › Table S1.pdf]

Table S1. Promising Zhong-derived wheat–*Thinopyrum intermedium* lines identified through combined evaluation of powdery mildew resistance, aphid response, and agronomic performance.

Line	Zhong background	APR pattern	Seedling-stage response	Aphid response	Resistance profile	Plant height (cm)	Spike length (cm)	Number of spikelet per spike	Number of grains per spike
S19	Zhong 2	MR	R	LR	Triple-resistant	95.8 ± 5.26	15.8 ± 1.79	18.4 ± 2.07	51 ± 9.14
S23	Zhong 2 and 5	SR	R	MR	Triple-resistant	106.2 ± 3.63	13.2 ± 1.64	16 ± 1.22	42.8 ± 7.6
S49	Zhong 5	MR	R	LR	Triple-resistant	111.2 ± 8.38	15 ± 0.71	17.8 ± 1.3	68.6 ± 13.83
S53	Zhong 2 and 5	SR	R	MR	Triple-resistant	112.2 ± 8.7	11.8 ± 0.45	15 ± 1.87	50.6 ± 10.6
S55	Zhong 2 and 5	SR	R	MR	Triple-resistant	100.6 ± 10.74	11.8 ± 1.09	17 ± 1.41	51.6 ± 4.98
S63	Zhong 2 and 5	SR	R	LR	Triple-resistant	107.2 ± 6.38	14.4 ± 1.14	17 ± 0.71	48.6 ± 8.44
S83	Zhong 5	SR	R	LR	Triple-resistant	115.8 ± 4.21	18 ± 1.22	19.8 ± 0.84	54.8 ± 7.19
S115	Zhong 5	SR	R	LR	Triple-resistant	109.8 ± 8.58	12.8 ± 1.92	18.4 ± 0.89	59.6 ± 4.51
S117	Zhong 5	SR	R	LR	Triple-resistant	122 ± 6.86	11.8 ± 0.84	16 ± 0.71	55.8 ± 6.38

Adult-plant powdery mildew responses were evaluated across three consecutive years. Agronomic trait values represent means across environments. The aphid assessment was conducted under natural field infestation conditions
